# Optimizing surgical margins in the treatment of eyelid Merkel cell carcinoma: a tertiary center experience and literature review

**DOI:** 10.3389/fopht.2025.1691372

**Published:** 2025-12-16

**Authors:** Diego Strianese, Claudio Barbato, Mario Troisi, Vittoria Lanni, Vincenzo Damiano, Rosa Maria Di Crescenzo, Maria Laura Passaro, Antonella D’Aponte, Raffaele Nubi, Manuel Conson, Dana Cohen, Giuseppe Mariniello, Ciro Costagliola, Adriana Iuliano

**Affiliations:** 1 Eye Clinic, Department of Neurosciences, Reproductive and Odontostomatological Sciences, University of Naples Federico II, Naples, Italy; 2 Department of Clinical Medicine and Surgery, University of Naples Federico II, Naples, Italy; 3 Division of Pathology, Department of Advanced Biomedical Sciences, University of Naples Federico II, Naples, Italy; 4 Department of Medicine and Health Sciences “V. Tiberio”, University of Molise, Campobasso, Italy; 5 Department of Advanced Biomedical Sciences, University of Naples Federico II, Naples, Italy; 6 Division of Neurosurgery, Department of Neurosciences, Reproductive and Odontostomatological Sciences, University of Naples Federico II, Naples, Italy

**Keywords:** eyelid neoplasm, immune checkpoint inhibitors, immunohistochemistry, Merkel cell carcinoma, Merkel cell polyomavirus, ocular adnexal tumor, periocular malignancy

## Abstract

**Background:**

Merkel cell carcinoma (MCC) of the eyelid is rare and aggressive. Diagnostic delay and inadequate excision may promote early nodal spread. We assessed the influence of surgical margins and re-excision timing on outcomes, supported by a PRISMA-guided systematic review on metastatic risk.

**Methods:**

A single-center retrospective series (2012–2024) included 9 histologically confirmed eyelid MCCs, analyzing presentation, treatment, and outcomes. Surgical strategies were classified as one-step wide local excision (1WLE, ≥5 mm), two-step wide local excision (2WLE) with early (E2WLE, ≤2 months) or late (L2WLE, 6 months) re-excision, and insufficient margin excision (IME, <2 mm without re-excision). A systematic review identified periocular MCC cases with individual-level data on margins and outcomes.

**Results:**

Patients (median age 71.8 years, range 42–92; 89% female) all presented with solitary nodules on the upper eyelid, and were node-negative and metastasis-free at diagnosis, consistent with AJCC 8th clinical stage I–IIA.Median follow-up was 48 months (IQR 12–120). Treatments included 1WLE (n=4), 2WLE (n=3; 2 E2WLE, 1 L2WLE), and IME (n=2). Three patients (33%) developed cervical lymph node metastases within 1–3 months: one after L2WLE (fatal at 12 months) and two after IME. Both IME patients showed marked responses to Avelumab. Of the remaining six, four (67%) remained disease-free and two (33%) died of unrelated causes. Metastatic risk was significantly higher after IME versus sufficient margins (p=0.0119). In the PRISMA-guided review (76 eyelid MCC), insufficient margins correlated with adverse outcomes; in a subset without baseline metastasis (n=39), insufficient margins increased risk of recurrence/metastasis (OR 10.56; 95% CI 1.84–77.24**;** Fisher’s exact p=0.002).

**Conclusion:**

In eyelid MCC, adequate margins at first surgery or early re-excision are crucial to prevent early nodal spread. Our findings emphasize the prognostic value of surgical adequacy and support incorporating wide excision into initial management. Further multicenter studies are warranted to define evidence-based management pathways, improve long-term outcomes, and clarify the role of checkpoint inhibition in periocular MCC.

## 1 Introduction

Merkel cells were first described by Friedrich Merkel in 1875 as clear, large, round cells in the basal epidermis (1). A century later, Toker et al. reported a rare, aggressive neuroendocrine skin cancer, now called Merkel cell carcinoma (MCC) (1). Although eponymously linked to Merkel cells, the tumor’s cell of origin remains debated: the B-cell theory is supported by B-cell marker expression in a subset of tumors (2), whereas the coexistence of MCC with squamous cell carcinoma favors an epithelial precursor in some cases (3). Most investigators thus consider mature Merkel cells an unlikely universal source (4).

MCC is uncommon and its incidence varies by geography. In the EU, the annual incidence was ~0.13/100,000 in 1995–2002 (5); in the United States, it is ~0.7/100,000 (6). Most lesions arise on chronically ultraviolet (UV)-exposed site; about 42.6% occur on the head/neck (6), and 2.5–10% involve the eyelid (5, 6). Recognized risk factors include advanced age, Caucasian ethnicity, cumulative UV exposure, and immunosuppression (e.g., HIV, transplant) (5, 6). In 2008, Feng et al. identified Merkel cell polyomavirus (MCPyV) clonally integrated in MCC (7), a finding replicated in ~80% of tumors and consistent with a key etiologic role (8). This viral *vs* UV-driven dichotomy carries clinical and biological implications: MCPyV-positive tumors may elicit antigen-specific immunity and can aberrantly express PD-L1, with potential relevance for immune checkpoint inhibition (9, 10). Clinically, periocular MCC often presents on the upper eyelid, near the margin, sometimes with madarosis, and is frequently misdiagnosed as chalazion/cyst or basal cell carcinoma (5, 11–14). Typical lesions are painless, rapidly enlarging, violaceous-red nodules, aligning with the AEIOU features (Asymptomatic, Expanding rapidly ≤3 months, Immunosuppressed, Older >50, UV-exposed site) (11, 15). Histopathology shows a poorly differentiated neuroendocrine carcinoma expressing cytokeratins (AE1/3, CAM5.2, CK20) and neuroendocrine markers (synaptophysin, chromogranin, NSE) (11, 12). A key differential is small-cell lung carcinoma: MCC is typically cytokeratin 20 (CK20)-positive and thyroid transcription factor-1 (TTF-1)-negative, whereas SCLC frequently expresses TTF-1 (12, 16).

Multiple staging systems have been used historically; contemporary series use AJCC TNM staging for eyelid carcinoma (17). Eyelid MCC appears to have more favorable local control and lower distant relapse than extra-ocular MCC when managed within specialized centers (17–19). Surgery with attention to margin adequacy and adjuvant radiotherapy forms the cornerstone of localized disease management (18). While 1–2 cm margins are standard for extra-facial MCC (19), Peters et al. reported effective local control of eyelid MCC with 5-mm margins, consistent with practices for other aggressive eyelid malignancies (20). For advanced or unresectable disease, immune checkpoint inhibitors (ICI) have demonstrated promising activity (21–23); Avelumab became the first FDA-approved therapy for MCC in 2017 (24), supporting further evaluation in periocular presentations where prospective data are still limited (21–24).

The aim of this study is to describe presentation, management, and outcomes of eyelid MCC in a tertiary center and to test the association between initial margin adequacy (≥5 mm *vs <*5 mm without timely re-excision) and metastasis/recurrence, and to examine whether initial margin adequacy predicts metastasis/recurrence in our cohort and in a contemporaneous systematic evidence synthesis.

## 2 Methods

### 2.1 Patients and study design

A non-comparative, retrospective case series was conducted at a single tertiary referral center from January 2012 to January 2024. Nine patients with Merkel cell carcinoma (MCC) of the eyelid were identified through institutional medical records, and data were collected retrospectively. All patients had a confirmed diagnosis with pathology results.

Inclusion criteria were: (1) confirmed diagnosis of MCC by immunohistochemistry, *(2)* primary localization of the tumor to the eyelid, *(3)* managed at our institution during the study period with complete baseline clinicopathologic data, and *(4)* availability of complete clinical records with a minimum follow-up of one year. Exclusion criteria included *(1)* eyelid involvement due to regional/distant metastasis from an MCC primary outside the periocular region, *(2)* absence of definitive histopathological confirmation, *(3)* prior definitive treatment elsewhere with undocumented details that prevent outcome attribution, *(4)* collision tumors where the MCC component cannot be analyzed independently, and *(5)* incomplete clinical records or insufficient follow-up information.

For each patient, the following data were collected: age at diagnosis, sex, initial presenting symptoms, anatomical origin of the lesion, presence of regional or distant metastases at diagnosis, type of primary treatment administered (surgical excision, adjuvant radiotherapy, and/or chemotherapy), margin status, recurrence (local or regional), development of distant metastases during follow-up, duration of follow-up, and overall survival. At diagnosis, all patients underwent regional examination and high-resolution lymph-node ultrasound (preauricular, parotid, levels I–V). Contrast-enhanced head–neck CT was obtained in all cases. Whole-body PET/CT was performed in patients with suspicious US/CT. Sentinel lymph node biopsy (SLNB) was not performed at the baseline as no regional or distant metastases were detected at presentation. Clinical staging was assigned according to the TNM staging system for Merkel cell carcinoma, AJCC Cancer Staging Manual, 8th Edition.

All surgical decisions were taken in a multidisciplinary setting (oculoplastic oncology, dermatopathology, radiation oncology), balancing oncologic control with eyelid function and reconstruction. Resections were planned as wide local excisions (WLE) with a predefined margin policy adapted to periocular anatomy. Our unit targets ≥5 mm gross margins. When anatomy or reconstruction constraints preclude this at first pass, we perform a planned two-step WLE: an initial diagnostic/excisional pass to remove the tumor with clear but <5 mm microscopic margins, followed by early re-excision (≤8 weeks) to extend margins to ≥5 mm once histology confirms MCC and reconstructive planning is optimized (E2WLE). Margin were defined sufficient as histologic tumor-free margins ≥5 mm, and insufficient in case of insufficient margin excision (IME) <5 mm.

The study adhered to the tenets of the Declaration of Helsinki and was approved by the Ethics Committee of the University of Naples Federico II. Owing to its retrospective design and use of anonymized data, the requirement for individual informed consent was waived. For any images or data with potential identifiability, specific written consent for publication was obtained. Systematic Review.

This systematic review was conducted in accordance with the Preferred Reporting Items for Systematic Reviews and Meta-Analyses (PRISMA) guidelines (25). Studies were considered eligible if they involved patients diagnosed with Merkel cell carcinoma. Given the rarity of long-term follow-up data in this population, no minimum follow-up duration was required for inclusion.

The inclusion criteria were *(1)* human patients with eyelid/periocular MCC, *(2)* Observational studies (prospective/retrospective cohorts, case–control, case series ≥3 cases, and single case reports) when they specifically report periocular MCC with clinical course/outcome, *(3)* histopathological confirmation with immunohistochemistry consistent with MCC, *(4)* any of the following—local/regional recurrence, nodal status and management (SLNB/therapeutic dissection), distant metastasis, margin status, adjuvant therapy (RT/systemic), disease-specific survival, overall survival, complications, *(5)* publications from January 2011 to December 2024, *(6)* English language, *(7)* any type of settings (single-center, multicenter, registry).

Exclusion criteria included *(1)* secondary analyses without primary patient-level data (e.g., reviews, meta-analyses, editorials, letters, technical notes), *(2)* abstract-only publications (conference abstracts/posters) without full data, *(3)* non-human, *in vitro*, or non-clinical studies, *(4)* studies with unconfirmed MCC diagnosis or insufficient immunophenotypic confirmation, *(5)* studies on non-periocular MCC without extractable periocular subgroup data, *(6)* duplicate/overlapping cohorts: when multiple publications reported the same or overlapping populations, we retained the most comprehensive dataset (largest sample and/or longest follow-up) or contacted authors when necessary.

A comprehensive literature search was performed using the Medline database, from January 2011 to December 2024. The following keywords and phrases were used in various combinations: “Merkel cell carcinoma,” “Primary cutaneous neuroendocrine carcinoma,” “Merkel carcinoma,” “eyelid,” and “head and neck.” A total of 113 records were identified through PubMed, and after screening and exclusion, 25 studies met the inclusion criteria and were included in the systematic review (8, 14, 26–48).

From each included study, the following data were extracted: first author and year of publication, number of patients, sex, age, presenting symptoms, anatomical site of the tumor, clinical stage, treatment approach, and clinical outcome. An initial descriptive analysis was conducted. Additionally, we extracted data from studies reporting individual-level details on Merkel cell carcinoma patients treated surgically, where tumor size, resection margin status, and clinical outcomes were clearly documented.

### 2.2 Statistical analysis

To investigate the potential association between surgical margin status and clinical outcomes, a statistical analysis was performed on the subset of patients for whom both variables were available. Patients were grouped based on resection margin status (sufficient *vs*. insufficient), and their outcomes were categorized as either positive (no recurrence or metastasis) or negative (recurrence and/or metastasis). The association between margin status and clinical outcome was assessed using Fisher’s exact test, and the strength of association was estimated by calculating the odds ratio (OR) with a 95% confidence interval. A p-value < 0.05 was considered statistically significant.

## 3 Results

A total of nine patients with histologically confirmed Merkel cell carcinoma (MCC) of the eyelid were identified from our institutional database. The median age at diagnosis was 71.8 years (range: 42–92), and the majority were female (n = 8, 88.9%). All patients presented with a single nodular lesion (n = 9, 100%) located on the upper eyelid (n = 9, 100%). At diagnosis—ultrasound and contrast-enhanced CT, with PET/CT when indicated—revealed no regional or distant metastases. Per AJCC 8th edition (clinical staging), three patients had Stage I disease and six had Stage IIA.

Individual data at presentation are detailed in **Table 1**. The median follow-up period was 48 months (IQR: 12–120).

**Table 1:** Demographic, clinical, radiologic, treatment, and outcome data for 9 patients with eyelid Merkel cell carcinoma managed at the Oculoplastic Unit, University of Naples Federico II (January 2012–December 2024).

Patient	Age	Sex	TNM	AJCC 8th edition clinical staging	Surgery	Initial margin status	Metastasis	Local recurrence	Systemic therapy	Follow-up duration (months)	Outcome
1	68	F	T2bcN0M0/T1N0M0	I	1WLE	≥5 mm	No	No	No	116	Alive
2	77	F	T3acN0M0/T3N0M0	IIA	1WLE	≥5 mm	No	No	No	120	Alive
3	68	F	T3acN0M0/T3N0M0	IIA	1WLE	≥5 mm	No	No	No	12	Alive
4	76	F	T2acN0M0/T1N0M0	I	1WLE	≥5 mm	No	No	No	120	Died for other reasons
5	42	F	T2bcN0M0/T1N0M	I	E2WLE	<5 mm ( early corrected)	No	No	No	12	Alive
6	85	M	T3acN0M0	IIA	E2WLE	<5 mm ( early corrected)	No	No	No	5	Died for other reasons
7	65	F	T1cN0M0/T2bcN0M0	IIA	L2WLE	<5 mm (late correction)	Yes (cervical + distant)	No	No	12	Died of MCC
8	73	F	T3aN0M0	IIA	IMR	<3 mm (not corrected)	Yes (cervical)	Yes	Avelumab	8	Alive with response
9	92	F	T3acN0M0	IIA	IMR	<3 mm (not corrected)	Yes (cervical)	No	Avelumab	8	Alive with response

Staging was performed according to the AJCC/TNM 8th edition clinical classifications (AJCC Cancer Staging Manual).

1WLE, One-Step Wide Local Excision (clinical margins ≥ 5 mm at index surgery), E2WLE, Early Second-step Wide Local Excision (completed within ≤ 2 months after initial margins < 5 mm), L2WLE, Delayed Second-Step Wide Local Excision (second-step WLE delayed ~5 months after a partial first excision), IMR, Insufficient Margin Resection (initial clinical margins < 3 mm with no second-step WLE performed).

Surgical excision was performed in all cases.

Four patients underwent one-step complete excision (1WLE) with margins ≥5 mm. Two patients (22%) underwent early (within 2 months) two-step wide local excision (E2WLE) with initial clear margins but less than 5 mm. One patient initially underwent partial excision elsewhere with close/positive margins and was referred to our unit. At ~6 months, we performed second-stage WLE (L2WLE) achieving ≥5 mm microscopic margins circumferentially (deep margin to 6.5mm), followed by skin graft. Two patients underwent initial local resection with clinical margins smaller than 2 mm but were unable to undergo a 2-step WLE re-excision (IME);. Three patients (33.3%) developed cervical lymph node metastasis (stage III), during a follow-up period ranging from 1 month to 3 month with one patient in the L2WLE margin group and two in the IME group. The patient with L2WLE developed distant metastases to the parotid gland and liver and succumbed to the disease approximately 12 months after diagnosis. Both patients in the IME group with metastatic disease were treated with Avelumab, exhibiting a remarkable response.

One of the two patients of the IME group had local recurrence and lymph node metastasis (14.3% of the cohort) within one month from the initial excision. Due to the patient’s age (92 years) and comorbidities, systemic therapy with Avelumab was initiated, resulting in regression of the lesion. The other metastatic patient of the IME group developed lateral cervical lymph node involvement approximately two months after surgery; the board recommended to commence the patient with Avelumab instead of proceeding with the second step wide excision, being the disease already disseminated. The patient responded to Avelumab with remarkable regression of the lymph node metastasis, which has been observed during 8-month follow-up.

**Figure 1:**
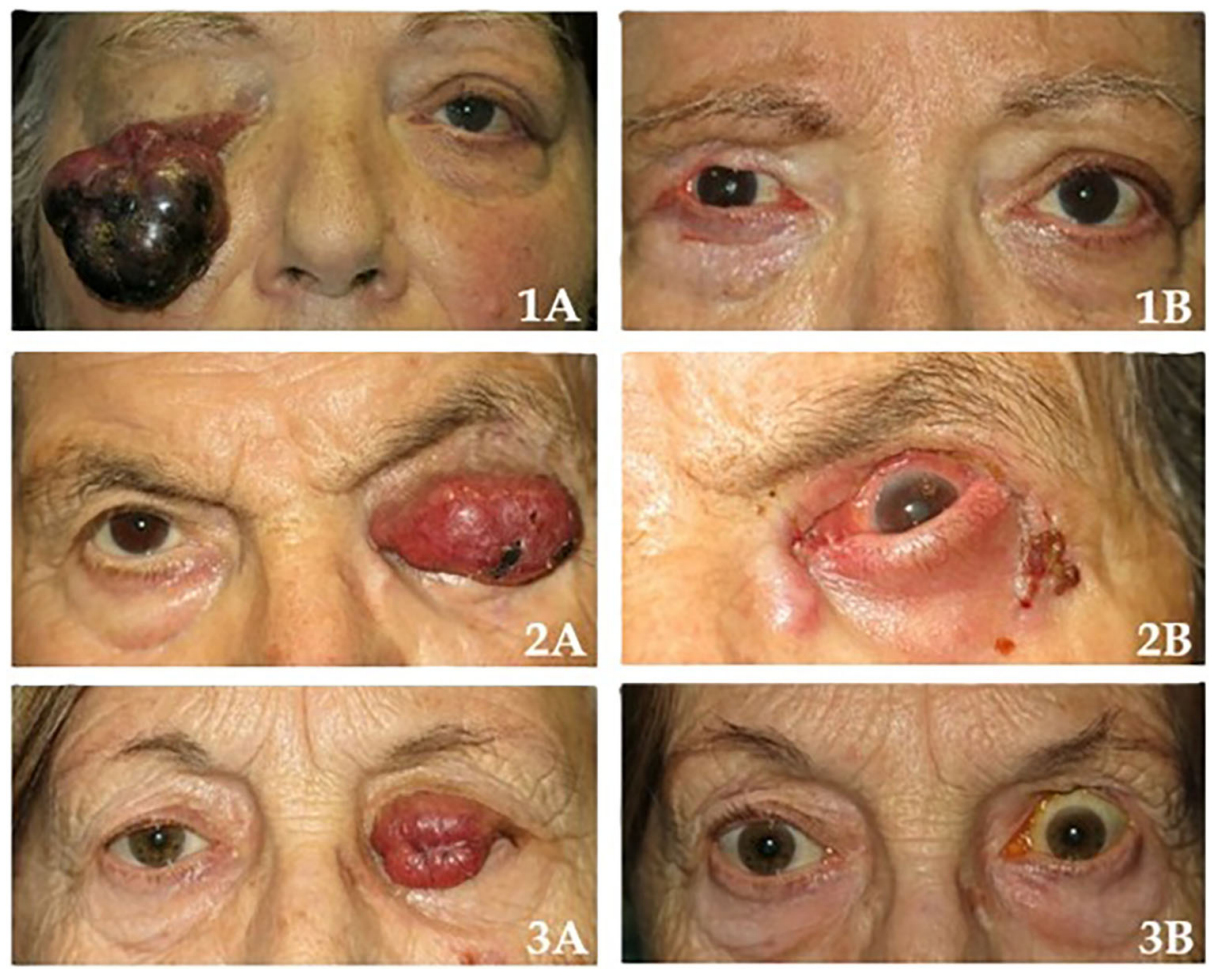
Case 1 presurgery (1A) and postsurgery (1B); case 2 presurgery (2A) and postsurgery (2B); case 3 presurgery (3A) and postsurgery (3B).

Of the remaining patients, four (57.1%) were alive without disease, and two (28.6%) had died from unrelated causes, free of disease until they passed away. A comparison of clinical outcomes between patients with initially sufficient versus insufficient surgical margins revealed a statistically significant difference in terms of metastatic disease (p-value = 0.0119). Clinical presentation and postoperative appearance of three representative cases of eyelid Merkel cell carcinoma are reported in **Figure 1**.

### 3.1 Literature review

**Figure 2:**
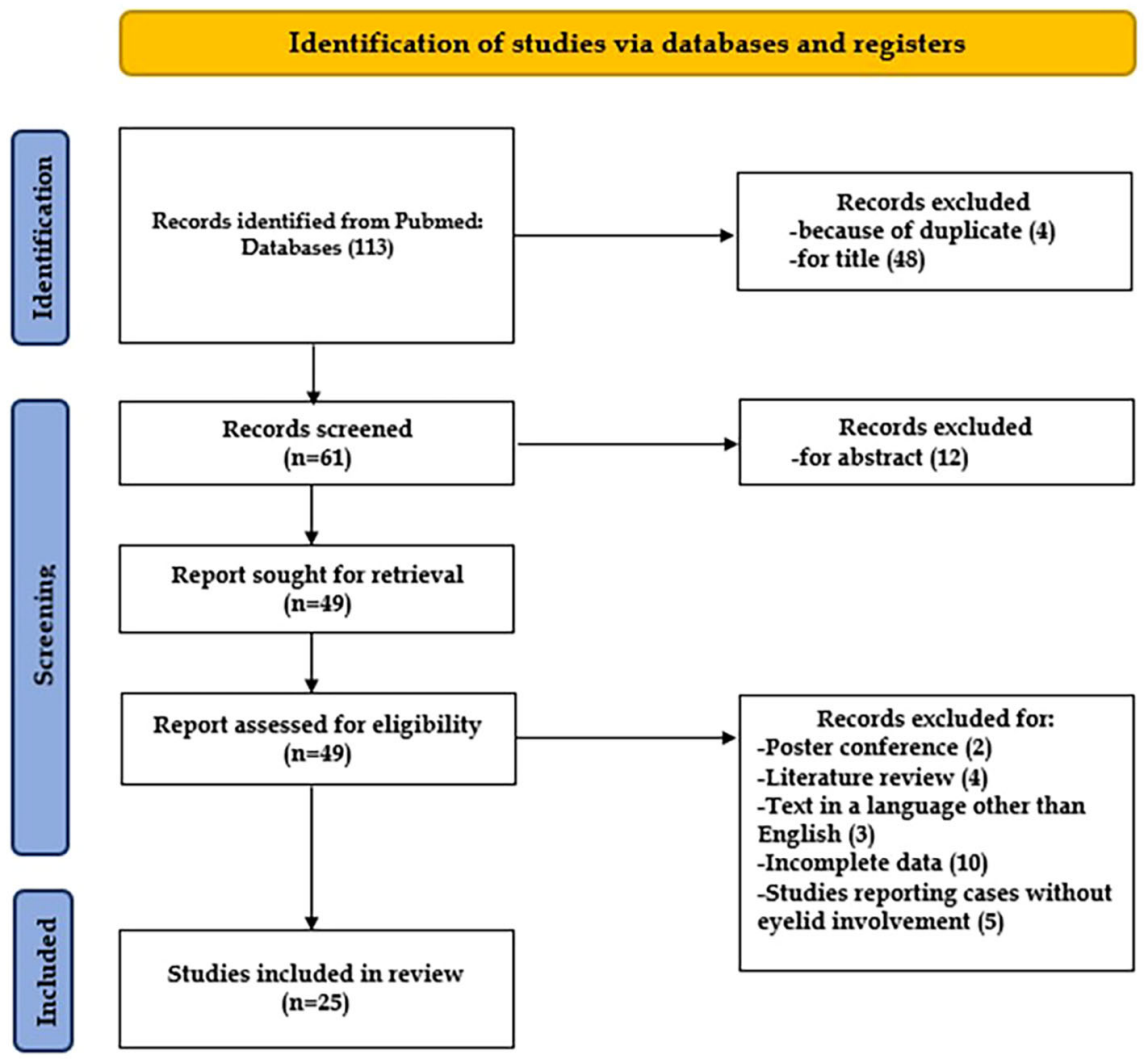
Flow chart showing the methods for the selection of the studies included in the review, following PRISMA (25).

After screening a total of 113 articles, 25 met the predefined inclusion criteria and were included in the final review, as illustrated in **Figure 2**. Overall, the review comprised 76 patients diagnosed with Merkel cell carcinoma (MCC) of the eyelid.

Data on patient sex were available for 75 out of 76 individuals (98.7%). Among them, 46 were female (61.3%) and 29 were male (38.7%). Presenting symptoms were reported in 94.7% of cases (n = 72/76), with the most common manifestation being a “single nodular lesion” (n = 60/72, 83.3%). Less typical presentations included “chalazion,” “single ulcerated lesion,” “tan cystic lesion,” or “multiple conjunctival nodules” (n = 12/72, 16.7%).

The anatomical origin of the tumor was reported for all patients. The most frequently involved site was the upper eyelid (n = 57/76, 75.0%), followed by the lower eyelid (n = 18/76, 23.7%). Metastatic disease at diagnosis was observed in 11 cases (14.5%), while the remaining 65 patients (85.5%) presented with localized tumors.

Full details of the included cases are summarized in **Table 2**.

**Table 2:** Demographic, clinical, and radiological characteristics of 76 eyelid Merkel cell carcinoma cases from a systematic review of the literature.

Authors/year		Number of cases (tot 76)	Sex, mean age (years)	Presenting symptoms	Anatomical origin	MCPyV+ /MCpyV-	Metastasis at the diagnosis
1	Sugamata et al., 2011 (26)	1	1 M (71 Years)	Nodular lesion 8x6mm	Lower eyelid	n.a.	NO
2	Santos et al. 2019 (27)	1	1 M (76 Years)	Nodular lesion 2-5cm (T2)	Lower eyelid	n.a.	YES
3	Boileau et al. 2023 (28)	11	4 M – 7 F (77 Years)	11/11 Nodular lesion Median size 13.2mm	10/11 Upper eyelid 1/11 Lower eyelid	n.a.	11/11 NO
4	Feng et al. 2021 (29)	1	1 M (91 Years)	Nodular lesion 32x30mm	Lower eyelid	n.a.	NO
5	Bostan et al. 2017 (30)	3	3 F (77 Years)	1. “Chalazion” 10x15mm 2. Nodular lesion 30x25mm 3. “Chalazion” 5x7mm	2/3 Upper eyelid 1/3 Lower eyelid	n.a.	3/3 NO
6	Grubb et al. 2021 (31)	1	1 F (71 Years)	Nodular lesion 30x20mm	Lower eyelid	MCpyV-	YES
7	Tran MN et al., 2022 (32)	1	1 M (73 Years)	Ulcerated lesion 20x20mm	Temporal Conjunctiva	n.a.	NO
8	Festa Kotelnikova et al., 2020 (33)	3	2 M – 1 F (74 Years)	n.a.	2/3 Lower eyelid 1/3 Upper eyelid	n.a.	1/3 YES 2/3 NO
9	Berkowitz MD et al., 2019 (34)	1	1 F (87 Years)	Tan cistic lesion >10mm	Upper eyelid	n.a.	NO
10	Chen et al. 2011 (35)	1	1 F (51 Years)	Ulcerated lesion 200x150mm	Upper eyelid	n.a.	YES
11	Zhu et al. 2024 (36)	1	1 F (79 Years)	Nodular lesion 20x18mm	Lower eyelid	n.a	YES
12	Zhan et al. 2023 (37)	1	n.a.	n.a.	Upper eyelid	n.a.	NO
13	Komatsu et al. 2024 (38)	1	1 F (37 Years)	Nodular lesion 12x10mm	Lower eyelid	n.a.	NO
14	Furdova et al. 2018 (39)	2	2 F (71 Years)	1. Nodular lesion 20x20mm 2. Nodular lesion 40x26mm	2/2 Upper eyelid	n.a.	1/2 YES 1/2 NO
15	Casey et al. 2021 (40)	1	1 F (52 Years)	Nodular lesion n.a	Upper eyelid	n.a	YES
16	Atamney et al. 2016 (41)	2	1 M – 1 F (89 Years)	1. “Chalazion” 10mm 2. “Chalazion” 2mm	2/2 Upper eyelid	n.a.	1/2 NO 1/2 YES
17	Riesco et al. 2016 (42)	5	2 M – 3 F (79 Years)	5/5 Nodular lesion Median size 25x21mm	3/5 Upper eyelid 2/5 Lower eyelid	n.a.	5/5 NO
18	Krasny et al., 2018 (43)	3	3 F (64 Years)	1. “Chalazion” 13x10mm 2. Nodular lesion 8x6mm 3. n.a.	3/3 Upper eyelid	n.a.	3/3 NO
19	Herbert et al. 2014 (14)	21	10 M – 11 F (77 Years)	12/21 Nodular lesion <20mm 9/21 Nodular lesion >20mm, <50mm	17/21 Upper eyelid 4/21 Lower eyelid	n.a.	19/21 NO 2/21 YES
20	Baccarani et al. 2013 (44)	1	1 F (73 Years)	Nodular lesion 40mm diameter	Upper eyelid	n.a.	NO
21	Komatsu et al. 2023 (8)	10	4 M – 6 F (79 Years)	10/10 Nodular lesion Median size 15.8mmx9.2mm	10/10 Upper eyelid	10/10 MCpyV+	10/10 NO
22	Yamanouchi et al., 2013 (45)	1	1 M (80 Years)	“Chalazion” 30mmx15mm	Upper eyelid	n.a.	NO
23	Hao et al. 2016 (46)	1	1 F (85 Years)	Nodular lesion 30x20mm	Lower eyelid	n.a.	NO
24	Shah et al. 2012 (47)	1	1 M (60 Years)	Multiple conjunctival nodules	Lower eyelid	n.a.	NO
25	Toto et al. 2019 (48)	1	1 F (71 Years)	“Chalazion” 41x16mm	Upper eyelid	n.a.	NO

MCPyV, Merkel cell polyomavirus, n.a., not available.

For the subset analysis—restricted to surgically treated patients with individual-level staging data—we excluded all cases with lymph-node involvement or distant metastasis at diagnosis to avoid immortal-time bias and reduce stage-related confounding, thereby isolating the potential association between surgical margin status and oncologic outcome. A total of 16 studies (39 patients) were included in this subgroup. The mean age was 74 years (SD 13), and the majority were female (49%), while 23% were male, and 28% had missing data on sex. Regarding clinical stage, 77% were stage I, and 23% were stage II. Margins were classified as sufficient in 69% of cases and insufficient in 31%. Adjuvant radiotherapy was administered in 53% of cases, and adjuvant chemotherapy in 5.3%. With regard to clinical outcomes, 69% of patients had a favorable course with no evidence of metastasis, whereas 31% developed metastatic disease during follow-up. Details are summarized in **Table 3**.

**Table 3:** Treatments and outcomes for 76 eyelid Merkel cell carcinoma cases from a systematic review of the literature.

Authors/year		Number of cases	Type of treatment	Local relapse	Metastasis relapse	Time of follow-up (months)	Status
1	Sugamata et al., 2011 (26)	1	S + RT + CHT	YES	YES	24	Alive
2	Santos et al. 2019 (27)	1	S	NO	YES	1	Died for dissemination
3	Boileau et al. 2023 (28)	11	2/11 EBRT 9/11 EBRT + LNI	11/11 NO	11/11 NO	62*	4/11 Alive 7/11 Died for other reasons
4	Feng et al. 2021 (29)	1	S + AP	NO	NO	27	Died for other reasons
5	Bostan et al. 2017 (30)	3	1/3 S 1/3 S + RT 1/3 S +S(LN) +LNI	1/3 YES 1/3 NO 1/ n.a.	1/3 YES 2/3 NO	36*	3/3 Alive
6	Grubb et al. 2021 (31)	1	S+LNI	NO	NO	6	Alive
7	Tran MN et al., 2022 (32)	1	S+RT+LNI+ CHT+ICI	YES	YES	10	Died for dissemination
8	Festa Kotelnikova et al., 2020 (33)	3	1/3 S 2/3 S + S(LN)	1/3 YES 2/3 NO	2/3 YES 1/3 NO	32*	3/3 Alive
9	Berkowitz MD et al., 2019 (34)	1	S + S(LN)	NO	YES	36	Alive
10	Chen et al. 2011 (35)	1	S	NO	NO	n.a.	n.a.
11	Zhu et al. 2024 (36)	1	S + S(LN)	YES	n.a	n.a	n.a
12	Zhan et al. 2023 (37)	1	ICI	n.a.	n.a.	n.a.	n.a.
13	Komatsu et al. 2024 (38)	1	S + RT + LNI	NO	NO	5	Alive
14	Furdova et al. 2018 (39)	2	2/2 S + S(LN) + CHT + RT	2/2 NO	1/2 NO 1/2 YES	18*	1/2 Died for dissemination 1/2 Alive
15	Casey et al. 2021 (40)	1	S + S(LN) + RT	NO	NO	96	Alive
16	Atamney et al. 2016 (41)	2	1/2 S 1/2 S + RT + LNI	2/2 NO	2/2 NO	21*	2/2 Alive
17	Riesco et al. 2016 (42)	5	3/5 S 1/5 S + LNI 1/5 S + RT + CHT	4/5 NO 1/5 YES	3/5 YES 2/5 NO	36*	1/5 died for dissemination 4/5 Alive
18	Krasny et al., 2018 (43)	3	2/3 S 1/3 S + CHT	2/3 YES 1/3 NO	3/3 NO	40*	1/3 Died for other reasons 2/3 Alive
19	Herbert et al. 2014 (14)	21	7/21 S 12/21 S + RT 1/21 S + CHT 1/21 S + S(LN) + CHT	19/21 NO 2/21 YES	15/21 NO 6/21 YES	54*	2/21 Died for dissemination 6/21 Died for other reasons 13/21 Alive
20	Baccarani et al. 2013 (44)	1	S + S(LN)	NO	NO	36*	Alive
21	Komatsu et al. 2023 (8)	10	6/10 S 1/10 S + S(LN) 3/10 S + RT	10/10 NO	2/10 YES 8/10 NO	50*	1/10 Died for other reasons 8/10 Alive 1/10 Died for dissemination
22	Yamanouchi et al., 2013 (45)	1	S + RT + LNI	NO	NO	24	Alive
23	Hao et al. 2016 (46)	1	S + RSI	YES	YES	30	Died for other reasons
24	Shah et al. 2012 (47)	1	CHT	NO	NO	7	n.a.
25	Toto et al. 2019 (48)	1	CHT + S	NO	NO	36	Alive

S, surgical excision of the primary tumor, RT, radiotherapy (external-beam unless otherwise specified), EBRT, external-beam radiotherapy, CHT, chemotherapy, LNI, lymph-node irradiation, S(LN), lymph-node surgery (sentinel node biopsy and/or lymphadenectomy), AP, adjuvant plaque brachytherapy (radioactive plaque), as reported in the source case, RSI, radioactive seed implantation (brachytherapy), ICI, immune checkpoint inhibitor therapy, n.a., not available.

*indicates the median follow-up time.

Among patients with available data on surgical margins and clinical outcome, we observed a statistically significant association between insufficient resection margins and negative clinical outcome. Specifically, patients with insufficient margins had a substantially higher risk of developing metastasis or recurrence compared to those with sufficient margins (Fisher’s exact test, p = 0.002). The odds ratio was 10.56 (95% CI: 1.84–77.24), indicating that the likelihood of a negative outcome was approximately ten times greater in patients with insufficient margins, regardless of tumor-size and T-stage.

## 4 Discussion

Our patient cohort exhibited a pronounced female predominance (8/9, 88.9%) and a mean age at diagnosis of 71.8 years, which aligns closely with the findings of our literature review (67.9% female among cases with known sex; mean age 74 years). Notably, all cases in our cohort involved the upper eyelid (9/9, 100%), consistent with published reports where 75% of lesions (57/76) were localized to this region. In contrast to the reviewed studies, where 14.5% (11/76) of patients presented with regional or distant metastasis at diagnosis, none of our patients exhibited metastatic disease at initial presentation.

The local recurrence rate in our cohort was 14.3% (1/7), mirroring the rate reported in the literature (14.9%, 11/74). Similarly, the proportion of patients who developed distant metastases (28.6%) was comparable to that observed in published reports (31.0%).

One of the primary challenges in managing Merkel cell carcinoma (MCC) of the eyelid is the frequent underestimation of the initial diagnosis. Given its rarity, MCC is often misdiagnosed as more common benign conditions, which can lead to delayed treatment and poorer outcomes. This challenge is well-documented in the literature (49), emphasizing the need for early recognition and appropriate surgical management to improve patient outcomes. In our cohort, one case had a partial resection of the lesion performed elsewhere due to an initial misdiagnosis of chalazion, which ultimately proved to be the only case that succumbed to the disease.

Clinical experience and data from the literature review underscore the importance of achieving adequate surgical margins (≥5 mm of free margin) during initial surgery to minimize the risk of local recurrence. Unlike many cases reported in the literature, where MCC of the eyelid is often misdiagnosed, leading to inadequate initial excision, all of our patients underwent immediate excisional biopsy at first presentation, except for one case. When clinical margins were <5 mm (4/9, 44.4%), surgical wide re-excision was promptly performed within two months in most cases. In our cohort, outcomes differed significantly by initial margin status: metastatic events were more frequent after insufficient versus sufficient margins (p = 0.0119). This pattern mirrors our PRISMA-guided systematic review, where insufficient margins were strongly associated with adverse outcomes (OR = 10.56; 95% CI, 1.84–77.24; p = 0.002). Notably, timely conversion to wide margins in our series appears to mitigate risk, reinforcing the concept that surgical timing is critical in eyelid MCC management (50, 51).

From a clinical perspective, our findings highlight the importance of considering MCC in the differential diagnosis of eyelid masses. The systematic adoption of diagnostic criteria such as the AEIOU acronym (asymptomatic/lack of tenderness, expanding rapidly, immune suppression, older than 50 years, and ultraviolet-exposed site on a person with fair skin) can facilitate early diagnostic suspicion. When clinical suspicion for MCC is high—based on AEIOU features such as rapid growth, lack of tenderness, and lash loss—a excisional biopsy with an early re-excision if needed is preferred over small incisional biopsy, minimizing tumor manipulation and expediting definitive margins. Furthermore, it is crucial to educate ophthalmic surgeons and dermatologists about the importance of ensuring adequate surgical margins during the initial excision, even when the definitive diagnosis has not yet been confirmed by histopathological examination.

Although Ki-67 quantification was not available for all patients, those who developed distant metastases had values exceeding 90%, whereas patients who remained disease-free had lower Ki-67 values. This suggests that Ki-67 may serve as a prognostic biomarker and inform decisions regarding adjuvant therapies. Further studies are needed to validate its role in clinical practice.

Notably, our most recent two patients received adjuvant Avelumab following early metastatic recurrence, and both demonstrated tumor regression and were alive after 8 months of follow-up. Although these observations align with published evidence supporting immune checkpoint inhibition in Merkel cell carcinoma, our small retrospective series cannot provide any conclusions regarding treatment efficacy. The therapeutic role of ICIs lies beyond the scope of this study, and definitive assessment requires larger, prospective oncologic datasets. Case reports have also described MCC arising *de novo* during PD-1 blockade, indicating that immunotherapy does not eliminate the risk of new primary tumors and that vigilant periocular surveillance remains essential (52). Overall, while ICIs have transformed systemic MCC management when surgery or radiation are constrained by function or stage (53), our findings do not allow disease-directed therapeutic recommendations. This study has several limitations, including the single-center cohort small sample size (n = 9), the retrospective nature, and incomplete data points, limited power for stage-adjusted analyses, possible selection bias in surgical strategy, and heterogeneity of adjuvant regimens. These constraints preclude causal inference and render our margin–outcome comparisons hypothesis-generating Despite these limitations, our findings emphasize the critical importance of early and well-coordinated surgical management in eyelid MCC (50, 51). Achieving adequate excision margins, either at the time of diagnosis or through timely re-excision, appears to be essential in minimizing the risk of recurrence and progression. Further multicenter studies are warranted to develop robust, evidence-based management guidelines and improve long-term patient outcomes, and clarify the role of checkpoint inhibition in periocular MCC.

## Data Availability

The original contributions presented in the study are included in the article/supplementary material. Further inquiries can be directed to the corresponding authors.
